# Gait Recognition Using Optical Motion Capture: A Decision Fusion Based Method

**DOI:** 10.3390/s21103496

**Published:** 2021-05-17

**Authors:** Li Wang, Yajun Li, Fei Xiong, Wenyu Zhang

**Affiliations:** 1 School of Physical Education, Sichuan Normal University, Chengdu 610101, China; 20060132@sicnu.edu.cn; 2 Department of Physical Education, Central South University, Changsha 410083, China; 3 School of Electronic and Information Engineering, Beijing Jiaotong University, Beijing 100044, China; xiongf@bjtu.edu.cn; 4 Institute of Artificial Intelligence, University of Science and Technology Beijing, Beijing 100083, China; wyzhang@ustb.edu.cn

**Keywords:** decision fusion, gait recognition, kernel ELM, optical motion capture, sensor fusion

## Abstract

Human identification based on motion capture data has received signification attentions for its wide applications in authentication and surveillance systems. The optical motion capture system (OMCS) can dynamically capture the high-precision three-dimensional locations of optical trackers that are implemented on a human body, but its potential in applications on gait recognition has not been studied in existing works. On the other hand, a typical OMCS can only support one player one time, which limits its capability and efficiency. In this paper, our goals are investigating the performance of OMCS-based gait recognition performance, and realizing gait recognition in OMCS such that it can support multiple players at the same time. We develop a gait recognition method based on decision fusion, and it includes the following four steps: feature extraction, unreliable feature calibration, classification of single motion frame, and decision fusion of multiple motion frame. We use kernel extreme learning machine (KELM) for single motion classification, and in particular we propose a reliability weighted sum (RWS) decision fusion method to combine the fuzzy decisions of the motion frames. We demonstrate the performance of the proposed method by using walking gait data collected from 76 participants, and results show that KELM significantly outperforms support vector machine (SVM) and random forest in the single motion frame classification task, and demonstrate that the proposed RWS decision fusion rule can achieve better fusion accuracy compared with conventional fusion rules. Our results also show that, with 10 motion trackers that are implemented on lower body locations, the proposed method can achieve 100% validation accuracy with less than 50 gait motion frames.

## 1. Introduction

Human identification using motion capture data has attracted much attention for its wide applications in authentication, surveillance, and medical applications [1]. Some commonly used identification methods, such as face recognition and fingerprint, are not preferred by some people who have sensitive privacy considerations [2]. Gait recognition does not require the privacy data of the target person, and therefore is one promising alternative that can be used for human identification. In addition, when the facial appearance of the target person is unavailable or changeable, it can be used for tracking the persons of interest in public security with the gait information of target persons [3].

In gait recognition, the system authenticates or classifies the target person by using her/his walking manner. The used gait data mainly include the following four modalities: camera image gait data [4], inertial acceleration sensor data [5], floor sensor data, and passive wireless signals [6] or wave radar data [7]. In addition, the recognition performance can be further improved by integrating the above four data modalities [8]. The optical motion capture systems (OMCSs), such as the Vicon [9], can obtain highly precise gait motion data of the target persons, and have been widely used in sports training [10], animation [11], medical analysis [12], and robotics [13]. For example, in sports training, using an optical motion capture system can accurately record the body motions, which is helpful for the coach and the athletes to analyze the characteristics of the motions, and provide new training guides for improving training performance [14]. Similarly, in medical analysis, the OMCSs can be used to capture the motion behavior of patients with Parkinson’s disease, and can be used as a diagnostic basis and in Parkinson treatment [15].

Existing gait recognition works mainly rely on sensor data or image data for classifying the object, and the recognition accuracy is not high enough for some applications with stringent accuracy requirements. The OMCSs can obtain high-precision gait motions, which may be helpful for improving the gait recognition performance, but its potentials in gait recognition has not been studied. Though it requires the implementation of optical motion trackers, the system does not require sensitive information like face image and fingerprint, thus it will be acceptable for some users with privacy concerns. In addition, OMCS is widely used in sports training, physical treatment, and visual effects production applications [11,12,13,14,15]. Existing work only support one player at one time, and it is necessary to develop gait recognition methods if we want to classify different persons in a multiple player scenario. In this way, the efficiency and capability of an OMCS can be greatly improved.

As such, in this paper we attempt to use an OMCS for human identification, and our goal is to achieve state of art identification accuracy with 10 optical trackers that are planted on the lower body locations of the target person, including thigh, lower leg, and foot, and both left and right body sides. The proposed method only requires the collection of several gait cycles of the participants, and the tracked body locations are chosen as the lower body part, which reduces the intrusion degree compared with other body parts such as chest and head. We propose a decision fusion-based gait recognition method, and compared with existing works, the proposed method has the following distinguished differences:

(1) We use an OMCS to record the gait trajectories of the persons and study the gait recognition problem with the obtained data, which has not been considered in existing works. Though OMCS requires the implementation of optical trackers, the system can obtain high-precision gait motion data, which is helpful to improve the classification accuracy when only using a relative smaller number of gait motions for human classification. In addition, the system does not require a large number of optical trackers, and using only 10 optical trackers can achieve very good classification accuracy, and even 100% accuracy when sample number is large enough.

(2) We use a powerful classifier, namely kernel extreme learning machine (KELM) [16], to conduct the classification process for the extracted feature data. Existing works mainly use SVM and random forest as the classifier [1]; in the considered problem of this paper, we show that KELM can achieve much higher classification accuracy and efficiency compared with the above two competitive classifiers.

(3) Instead of focusing on improving the classification accuracy of the base classifier, in this paper we highlight the importance of decision fusion of multiple motion frames, and we develop a first-classification-then-fusion method to achieve better classification performance, and the design of the decision fusion rules plays a vital role in improving the fusion performance. More specifically, we first conduct the classification process for each single motion frame, and then propose a reliability weighted sum (RWS) rule for combining the classification decisions of multiple motion frames. In the proposed RWS rule, we first transform the outputs of the KELM classifier into fuzzy decisions by using a membership transformation function, then compute the consistency matrix of all the fuzzy decisions by using a consistency degree measurement. With the obtained consistency matrix, we then use the Eigenvalue decomposition method to obtain the weight vector of the fuzzy decisions. A fuzzy decision with larger average consistency value to other fuzzy decisions means that it is more reliable, and the corresponding reliability weight value will be relatively larger, otherwise it will be relatively smaller.

(4) The experimental results on the collected gait motion data with 76 participants demonstrate that the proposed rule outperforms several decision rules, including sum rule, belief rule, weighted belief rule, product rule, and majority voting rule. The results also show that the proposed method can achieve 100% classification accuracy with 10 motion optical trackers, which shows its potentials for its applications in authentication and multiplayer motion tracking applications. 

The remainder of this paper is organized as follows: Section 2 gives a brief review to related work, the detailed process of the proposed decision fusion-based gait recognition method is shown in Section 3. Experimental results are shown in Section 4. Section 5 concludes the paper.

## 2. Related Work

Gait recognition has been extensively studied in for its wide applications in authentication, surveillance, training, and medical treatment [1,2,3]. In most existing methods, the gait recognition methodology mainly includes the following four steps: feature extraction, dimension reduction, and classification [1]. The gait motion data contains both the locations and velocity data; thus, it is more related to image gait data and acceleration sensor data. From the perspective of tracker implementation way, the acceleration sensor data is similar to the optical motion tracker used in this paper, since they all need to collected from the tracker sensors deployed on different locations of the human body. The difference is that accelerator cannot track the 3D positions of the gait, and it requires the system to collect sufficient data samples (e.g., more than 1000) to achieve a relative better classification performance [17]. For the optical motion tracker based system, its deployment cost is higher compared with inertial sensor system, but it can obtain high-quality and precise trajectories of the optical trackers. Since the precise gait motion trajectories are recorded, the proposed system can achieve good performance with very limited number of data samples or a few gait cycles. On the other hand, the image gait data can be regard as a 2D rejection of the 3D gait motions, and a set of gait images contain the trajectories of the monitored person. However, due to the limitation of image resolution, it is hard to extract precise 3-D precise trajectories from the image data without the assistance of range sensor, which decreases the recognition accuracy of the system [18].

In the feature extraction step, the system needs to extract expressive feature representations from the raw data. Although the quality of the extracted features has significant impacts on the classification accuracy of the gait recognition task, yet it is still not unified that which feature is the best choice that suits all situations. For image data, existing feature extraction methods can be categorized as model-based methods and model-free methods [1]. In model-based feature extraction, the features depicting the human body characteristics are extracted, typical features including stride length and step frequency [19], body distances [20], gait template [21], and velocity Hough transform [22]. According to the summary of Tang [23], the commonly used for extracting the acceleration data feature mainly include mean value, standard deviation, range, signal energy, spectral entropy, bandpower, etc. Some other features, including number of zero crossings, inter quartile range, average peak length, spectral edge frequency, are also used in gait recognition systems [24]. In this paper, we will also use relative distance feature and velocity features as in the input of the classifiers, which belongs to the methodology of model-based feature extraction. It has been shown that using relative distance of the body locations can achieve good classification performances since they are able to depict both body shape and motion actions [20]. In addition, experiments have shown that the walking speed is highly correlated to the age and gender of the target persons [25], thus it is also adopted in the proposed gait recognition method.

In the classification step, the most commonly used classifiers include support vector machine (SVM) [26], random forest (or Decision Tree Ensemble Classifier) [27]. The deep learning based method are also popular for gait recognition, such as deep convolutional neural networks (CNN) [28], deep autoencoder-decoder [29], and long short-term memory (LSTM) network [30]. In this paper, we want to train the classifier with only a limited training data; thus, deep learning methods are not suitable in this situation since they need large enough data to train the model. We will use a kernel extreme learning machine (KELM) classifier to conduct the gait recognition process for single frame motion data, and we will prove its superiority in classification accuracy compared with SVM and random forest. 

We emphasize that the gait recognition problem in this paper is different from the above existing works, in which the classifier usually requires a very large training data samples, and each sample is composed by several gait cycles to achieve good classification performance. In the proposed method, we only record several gait cycles for training the classifier for single motion classification, and 1–2 gait cycles for classification, which is efficient for data collection process. However, the available number of gait motion data is quite limited and cannot be solved by methods that require a larger number of training and test data. To fully use the decisions of each single motion frame, in this paper we first propose a method that first conducts frame-level classification, and then develop a decision fusion method to combine the decisions of the single frames, while in existing work the decision is directly made by the output of the classifier, and each classification requires a bunch of gait motion data frames. In the following section, we will illustrate the detailed steps of proposed gait recognition method using decision fusion.

## 3. Decision Fusion Based Gait Recognition

### 3.1. The Gait Motion Tracking System

**Figure 1:**
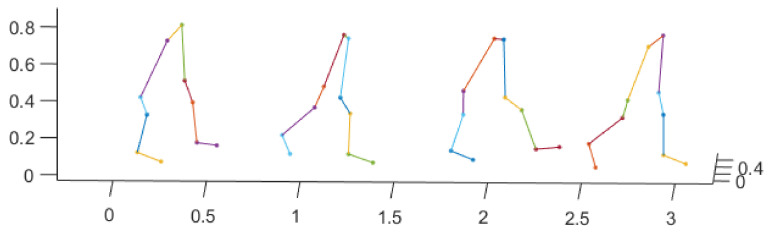
An example of the 3D gait motion track data.

We use optical motion trackers to record high-precision body motion trace to identify the target person. Before identification, the participants are required to wear a set of optical trackers, and then naturally and straightly walk through a flat test field with a length less than 5 m, which can be done in a very short period of time. There are 76 participants in total, with 46 females and 30 males, and their ages range from 20 to 60. The heights of the participants range from 144 to 178 cm, and the weights ranges from 42 to 115 kg. The sampling frequency of the body locations is 5 Hz, and there are 10 lower-body locations are recorded in each frame, which include thigh, knees, shin, ankle, and tiptoe, and both left and right sides are covered. The obtained walking lengths of the participants are different, which range from 2.37 to 4.15 m. An example of the recorded gait data is shown in Figure 1, in which 5 gait motion samples of one participant is plotted. Visually, we can see that it is hard to identify the target person without using a proper gait recognition method.

**Figure 2:**
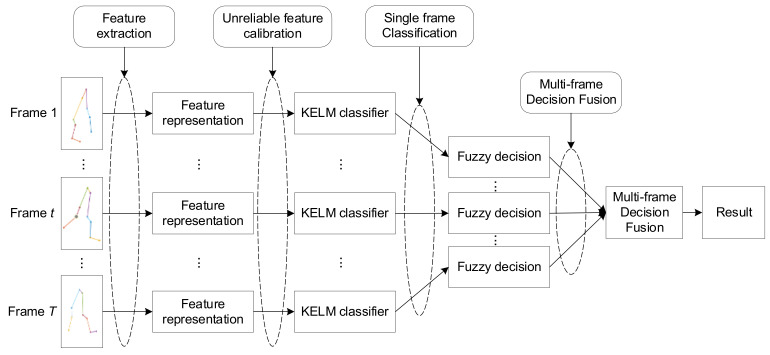
The flowchart of the proposed gait recognition method.

In this paper, we consider a gait recognition problem using high-precision optical gait motion trackers. Before identifying the target, the system previously prepared a set of training dataset with N different persons, denoted as $T=\left\{\left\{\left(x_{1},y_{1},z_{1}\right),c_{1}\right\},\ldots ;\;\left\{\left(x_{N},y_{N},z_{N}\right),c_{N}\right\}\right\}$, where $x_{i}=\left\{x_{i,1},\ldots ;x_{i,10}\right\}$, $y_{i}=\left\{y_{i,1},\ldots ;y_{i,10}\right\}$, and $z_{i}=\left\{z_{i,1},\ldots ;z_{i,10}\right\}$ denote the recorded 3D coordinates of the trackers of person i, and $c_{i}$ denotes its label. Note that the training data must at least contain a complete walking cycle of the person. Given a new person to be identified, the system record *T* consecutive motion frames, denote as $X=\left\{\left(x^{\left(1\right)},y^{\left(1\right)},z^{\left(1\right)}\right),\ldots ;\left(x^{\left(T\right)},y^{\left(T\right)},z^{\left(T\right)}\right)\right\}$, where $x^{\left(t\right)}=\{x_{1}^{\left(t\right)},\ldots ;x_{10}^{\left(t\right)}\}$, $y^{\left(t\right)}=\{y_{1}^{\left(t\right)},\ldots ;y_{10}^{\left(t\right)}\}$, and $z^{\left(t\right)}=\{z_{1}^{\left(t\right)},\ldots ;z_{10}^{\left(t\right)}\}$ denote the 3D coordinates of t-th gait motion frame. When the classifier is properly trained, our problem becomes identifying the target person according to the input motion data X. As shown in Figure 2, the proposed first-classification-then-fusion method mainly includes the following four steps:

(1) **Feature exaction**: For each person, the input raw data includes 10 motion tracks, and they cannot be directly used to classify the target object. In this paper, we want to identify the target person with a short gait motion capture trace; extracting the features from a trace recorded from motion tracker time series is not practical in this situation since each person may only has several gait cycles, and the number of collected gait motion is quite limited. As such, a feature exaction process that only extracts relative location distance and speed features from single frame data is proposed to obtain an expressive feature representation of the input data, and then the extracted features will be the input of the following identification process.

(2) **Unreliable feature calibration**: Though the OMCS can record high precise gait trajectory data, we observe that a recorded motion instance may be biased due to sensing failure or noise interference, and the corresponding the features of the biased motion data are also unreliable. Therefore, it is necessary to detect and calibrate the unreliable feature data, and relief their impact on the classification performance.

(3) **Classification**: In this paper, we will use a kernel extreme learning machine (KELM) to deal with the classification task for the feature data of each single gait motion frame. We will provide performance comparison results to demonstrate the advantages on classification accuracy and efficiency of KELM for the gait classification in the experimental section.

(4) **Decision fusion**: The motion frame number of different persons are also different due the variation of walking speed of the target person. With the obtained KELM outputs of all the frames, we then need to combine them into a unified one to obtain the final global decision. Compared with single frame classification, we expect a classification accuracy improvement after combining the decision of multiple frames, and the decision fusion rule will play a vital rule on the final fusion accuracy. 

In the next subsections, we will give a detailed illustration of the above four steps, along with their mathematical models.

### 3.2. Feature Extraction

Given a gait motion data, we use the relative distance of the tracker locations as the feature of the input motion data. The reason is that the relative distance metric can depict both walking action and physical body shape characteristics, which can effectively distinguish the differences between gait motions of two persons. In frame (*t*), for two trackers i,j with coordinates $\left(x_{i}^{\left(t\right)},\ldots ;x_{i}^{\left(t\right)}\right)$ and $\left(x_{j}^{\left(t\right)},\ldots ;x_{j}^{\left(t\right)}\right)$, their Euclidean distance can be computed by

(1) $$d_{i,j}^{\left(t\right)}=\sqrt{{\left(x_{i}^{\left(t\right)}-x_{j}^{\left(t\right)}\right)}^{2}+{\left(y_{i}^{\left(t\right)}-y_{j}^{\left(t\right)}\right)}^{2}+{\left(z_{i}^{\left(t\right)}-z_{j}^{\left(t\right)}\right)}^{2}}$$

In this way, we can obtain a pairwise distance matrix $D={\left[d_{i,j}\right]}_{\left\{10\times 10\right\}}$ that contains the relative distance metrics between the 10 optical tackers, in which $d_{i,i}^{\left(t\right)}=0$ and $d_{i,j}^{\left(t\right)}=d_{j,i}^{\left(t\right)}$. Since $D^{\left(t\right)}$ is symmetrical, and diagonal elements are all 0, we only use the elements of upper triangle or lower triangle of matrix $D^{\left(t\right)}$ as the feature representations. Let $d^{\left(t\right)}=\{d_{1}^{\left(t\right)},\ldots ,d_{K_{d}}^{\left(t\right)}\}$ be the one-dimensional vector that contains all the elements of upper triangle matrix of **D**, then we can know the feature dimension is $K_{d}=10\times 9/2=45$.

It has been shown that gait speed can be used as features for human identification and age prediction [23], thus except the relative distances among the optical trackers, we also include the x-axis speed of each tracker into the features vector. For tracker i, the x-axis coordinates in frames $\left(t\right)$ and $\left(t+1\right)$ are $x_{i}^{\left(t+1\right)}$ and $x_{i}^{\left(t\right)}$, then we can estimate the x-axis velocity as follows:

(2) $$v_{i}^{\left(t\right)}=\frac{1}{\tau }\left(x_{i}^{\left(t+1\right)}-x_{i}^{\left(t\right)}\right)$$

where τ denotes the time period between two consecutive gait motion frames. In this paper, τ=0.2 s. In this way, we can obtain a velocity feature vector $v^{\left(t\right)}=\{v_{1}^{\left(t\right)},\ldots ,v_{Kv}^{\left(t\right)}\}$, and $K_{v}=10$. Combined with the relative distance features, we finally obtain the gait feature vector of motion frame $\left(t\right)$ as $z^{\left(t\right)}=\{d^{\left(t\right)};v^{\left(t\right)}\}$. Note that, before the classification step, the features obtained need to be normalized since their magnitudes are different.

### 3.3. Unreliable Feature Calibration

Due to sensing failure or noise interference, the obtained track data may be biased, and even become outliers. Accordingly, the obtained features also will be unreliable, and will cause negative impacts on the classification performance of the gait recognition task; thus, the unreliable features need to be detected and calibrated. In this paper, we use a hypothesis test method to identify the unreliable features, in which we first estimated the probability density function (PDF) of each feature, then find the unreliable features that is larger or lower than the probability thresholds. Since the feature is irregularly distributed, and cannot be reasonably depicted by one specified distribution, thus we use the kernel density estimation method to estimate the PDFs of the features. Given a feature vector $z=\left\{z_{1},\ldots ,z_{L}\right\}$, where L denotes the number of feature data. The estimated PDF at point z is estimated as follows [31]

(3) $$f\left(z\right)=\frac{1}{L}\overset{L}{\underset{i=1}{\sum }}K\left(\frac{z-z_{i}}{\rho }\right)$$

where ρ>0 denotes that kernel parameter. In this paper, we use radial basis function as the kernel function, i.e., $K\left(\frac{z-z_{i}}{\rho }\right)=\exp(-\frac{{\left(z-z_{i}\right)}^{2}}{\rho })$, and the bandwidth parameter ρ is computed by $\rho =\sigma {\left(\frac{4}{3L}\right)}^{0.2}$ [32]. With the obtained PDF, we then can obtain the corresponding cumulative distribution function (CDF) by $\Phi \left(z\right)=\int_{o}^{z}p\left(z\right)dz$. Let $P_{th}$ be the probability threshold that decides whether the feature is unreliable or not. In other word, a feature data z will be regarded as unreliable when it satisfies the following condition:

(4) $$\Phi \left(z\right)>P_{th}\;\mathrm{or}\;\Phi \left(z\right)<1-P_{th}$$

The above criterion requires us to compute the CDF $\Phi \left(z\right)$ every time, which is not efficiency enough. We can also compute the corresponding upper bound $z_{ub}$ and lower bound $z_{lb}$ with respect to $P_{th}$ and $1-P_{th}$ by $z_{ub}=\Phi^{-1}\left(P_{th}\right)$ and $z_{lb}=\Phi^{-1}\left(1-P_{th}\right)$ respectively. In this way, expression (4) is equivalent to

(5) $$z>\Phi^{-1}\left(P_{th}\right)\;\mathrm{or}\;z<\Phi^{-1}\left(1-P_{th}\right)$$

For a feature data $z_{i}^{\left(t\right)}$, if it is judged as unreliable, in this paper we simply calibrate it as follows $z_{i}^{\left(t\right)}=z_{i}^{ub}$ if $z_{i}^{\left(t\right)}>\Phi^{-1}\left(P_{th}\right)$, and $z_{i}^{\left(t\right)}=z_{i}^{lb}$ if $z_{i}^{\left(t\right)}<\Phi^{-1}\left(1-P_{th}\right)$. Note that we do not delete the unreliable because the remained features are still reliable, and a classifier that is strong enough may still can classify the target with calibrated data. In the following multiple frame decision fusion process, the soft decision of a calibrated motion data is still useful for recognizing the target person.

**Figure 3:**
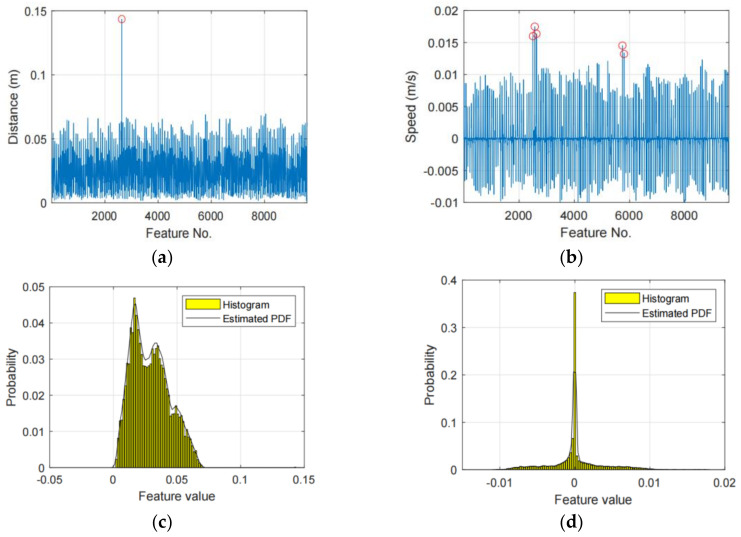
Two examples of unreliable feature, in which (**a**,**b**) plot the tracker distance features and gait speed, respectively, the unreliable features are marked with red circles, (**c**,**d**) plot the distribution of (**a**,**b**), respectively.

Figure 3 shows an example of the detected unreliable features, in which (a) and (b) plot the tracker distance features and gait speed respectively, (c) and (d) plot the PDFs of (a) and (b), respectively. When the probability threshold $P_{th}$ = 99.9%, we will find 1 and 5 outliers in (a) and (b), respectively, and the outlier points are marked with red circles. In this situation, the corresponding features will be to calibrated as their mean values to relief their impacts on the classification performance.

### 3.4. Classification with Single Gait Motion Frame

With the obtained features, we then use a pattern recognition classifier to identify the target person. The used classifier can be any proper classifier with acceptable classification performance, such as the commonly used support vector machine (SVM), and random forest. In the proposed decision fusion based gait recognition method, the classification performance of each single gait motion frame has critical influences on the following fusion accuracy, thus it is necessary to find a classifier with powerful classification capacities. However, from the test results, we found that the classification performances of SVM and random forest are not satisfactory to us. In this paper, we use KELM as the base classifier for its outstanding classification performance in both accuracy and efficiency. Let Z be the features extracted from training motion data, then we can obtain the corresponding kernel Gram matrix $K={\left[K_{i,j}\right]}_{M\times M}$ by using the kernel function, in which $K_{i,j}$ is computed by

(6) $$K_{i,j}=K\left(z_{i},z_{j}\right)$$

where $K\left(z_{i},z_{j}\right)$ denotes the kernel function with inputs $z_{i}\;\mathrm{and}\;z_{j}$. In this paper we use Gaussian kernel function, and $K_{i,j}$ is computed by

(7) $$K_{i,j}=e^{\frac{{\left(z_{i}-z_{i}\right)}^{2}}{h}}$$

where h>0 denotes the bandwidth function, usually it can be selected from $\left\{2^{-9},\ldots ,2^{0},\ldots ,2^{9}\right\}$. In the training process of KELM classifier, our goal is to obtain the output weight matrix β, which is computed by [33]

(8) $$\beta ={\left(K+\frac{1}{\lambda }\right)}^{-1}C$$

where λ denotes the regularization parameter, and it can be selected from $\left\{10^{-9},\ldots ,10^{0},\ldots ,10^{9}\right\}$. Note that parameters h and λ can be set by different trials, and the values can be chosen as the one with maximal classification performance. Now, given a new input feature data $z^{\left(t\right)}$, we can obtain the corresponding output of the KELM classifier as follows:

(9) $$o^{\left(t\right)}=K{\left(z^{\left(t\right)},Z\right)}^{T}\beta =K{\left(z^{\left(t\right)},Z\right)}^{T}{\left(K+\frac{1}{\lambda }\right)}^{-1}C$$

Note that $o^{\left(t\right)}$ is a vector that contains continuous predictions of target data, and we need an additional decision making process to if we want to know the final discrete predicted labels, namely the hard decisions.

**Remark** **1.** *A relative larger value of a KELM output means the probability that the target belongs to the corresponding class will be higher. Apparently, a class with maximal output will be regarded the hard decision of the classifier. For a KELM classifier, if an output $o_{i}^{\left(t\right)}$ is closer to −1, then it is more probable that the target does not belong to the corresponding class $c_{i}$. On the other hand, if $o_{i}^{\left(t\right)}$ is closer to 1, then it will be more probable that it belongs to the class $c_{i}$.*

Since we want to combine the decisions of the multiple motion frames, the output will be transformed to fuzzy decisions, and details will be introduced in the following subsection.

### 3.5. Decision Fusion of Multiple Motion Frames

To combine the decisions of the consecutive motion frames, in this paper we propose a reliability-weighted sum rule (RWS) that adjusting the fuzzy decisions by considering the differences among the fuzzy decisions. In general, a decision is relatively more consistent to other decisions, it is more reliable, otherwise more unreliable. In RMS, the obtained output data of KELM classifier are first transformed to fuzzy decisions by using a fuzzy membership function. More specifically, for frame t, the fuzzy membership that the target person belongs to class $c_{i}$ is defined as follows:

(10) $$\mu_{i}^{\left(t\right)}=\exp\left[-{\left(\frac{o^{\left(t\right)}}{\bar{o}+\gamma \sigma_{o}^{\left(t\right)}}\right)}^{2}\right]$$

where $\bar{o}$ and $\sigma_{o}^{\left(t\right)}$ denote the average value and the standard deviation of the $o^{\left(t\right)}$. Parameter γ is used for adjusting the discriminative degree of the obtained membership values. A larger value of γ will produce a larger span of the membership, and the discriminative degree is also higher. In this paper, we set γ=0.5 in default.

**Remark** **2.** *The above fuzzy decision means that, for one classifier output vector, a relative larger output of one class will be transformed to a larger membership compared with other classes, otherwise it will be relatively smaller. In particular, in KELM, we set the class label that the person belongs to as +1, and other class labels as −1. For example, in a classification task with 5 classes, the decision label vector when the person belongs to class 2 is $c={\left[-1,+1,-1,-1,-1\right]}^{T}$. Given a new gait instance that belongs to class 2 and suppose its output vector is $o={\left[-0.9,\;0.2,-0.4,-0.8,-0.95\right]}^{T}$, then the fuzzy decision computed by Equation (11) will be $\mu =\left[0.0005,0.6913,0.2284,0.0027,0.0002\right]$, we can see that a relative larger output value will produce a larger value of fuzzy membership.*

With the above membership transformation process, we can obtain the fuzzy decisions of all the motion frames, which are denoted as $\mu^{\left(1\right)},\ldots ,\mu^{\left(T\right)}$. One can directly combine the fuzzy decisions by using some classical decision fusion rules, such as the sum rule, product rule, and majority voting rule [34]. However, the reliabilities of the decisions are not considered in the above rules, which may decrease the accuracies of the final fusion results. If we can know a reasonable reliability for each fuzzy decision, then the impacts of the misclassified decisions can be reduced, and the classification accuracy of the global fusion results can be improved.

As such, in this paper we propose a reliability estimation method by using the consistency degrees among the fuzzy decisions. In belief function theory, the consistency degree between two basic belief assignments (BBAs) $m_{1}=\left\{m_{1}\left(\omega_{1}\right),\ldots ,m_{1}\left(\omega_{M}\right)\right\}$ and $m_{2}=\left\{m_{2}\left(\omega_{1}\right),\ldots ,m_{2}\left(\omega_{M}\right)\right\}$ is defined as follows [34]

(11) $$\varphi \left(m_{1},m_{2}\right)=\underset{\omega_{i}\cap \omega_{j}\neq \emptyset }{\sum }m_{1}\left(\omega_{i}\right)m_{2}\left(\omega_{j}\right).$$

Following the above definition, we define the consistency degree between two fuzzy decisions $\mu^{\left(1\right)}=\{\mu_{1}^{\left(1\right)},\ldots ,\mu_{M}^{\left(1\right)}\}$ and $\mu^{\left(2\right)}=\{\mu_{1}^{\left(2\right)},\ldots ,\mu_{M}^{\left(2\right)}\}$ as follows:

(12) $$\psi (\mu^{\left(1\right)},\mu^{\left(2\right)})=\underset{\omega_{i}\cap \omega_{j}\neq \emptyset }{\sum }\mu_{i}^{\left(1\right)}\mu_{j}^{\left(2\right)}.$$

Since we do not have compound classes in the above Equation, thus the consistency degree equals to the inner product of $\mu^{\left(1\right)}$ and $\mu^{\left(2\right)}$, as given by

(13) $$a_{1,2}=\psi (\mu^{\left(1\right)},\mu^{\left(2\right)})=\overset{M}{\underset{i=1}{\sum }}\mu_{i}^{\left(1\right)}\mu_{i}^{\left(2\right)}=<\mu^{\left(1\right)},\mu^{\left(2\right)}>$$

If the obtained consistency value $\psi \left(\mu^{\left(1\right)},\mu^{\left(2\right)}\right)$ is relative larger, then we can know that $\mu^{\left(1\right)}$ and $\mu^{\left(2\right)}$ are more similar with each other, otherwise they are more conflicting with each other.

**Remark** **3.** *For a complex classification task with multiple classes, there may exist several outputs with relative larger fuzzy membership values. For example, for a fuzzy decision $\mu^{\left(1\right)}={\left[0.1,0.1,\;0.7,\;0.85,\;0.1\right]}^{T}$, in which the memberships of class $c_{3}$ and $c_{4}$ are much larger than the other 3 classes, and both of them are probable to be results. Given another output vector $\mu^{\left(2\right)}={\left[0.1,0.1,\;0.2,\;0.85,\;0.1\right]}^{T}$, in which the membership of class $c_{4}$ is much larger than other classes. If the target belongs to class $c_{3}$, we can see that, when the reliability weights of the two fuzzy decisions are the same, fuzzy decision $\mu^{\left(2\right)}$ will impose a higher negative impact to the final decisions compared with $\mu^{\left(1\right)}$. As such, it is necessary to allocate a relative smaller reliability weight to $\mu^{\left(2\right)}$ to avoid misclassification risks.*

**Remark** **4.** *According to the above definition of decision consistency degree, we can see that, for two fuzzy decisions $\mu^{\left(1\right)}$ and $\mu^{\left(2\right)}$, if $\mu^{\left(1\right)}≽\mu^{\left(2\right)}$, i.e., the memberships of $\mu^{\left(1\right)}$ are all larger than the corresponding memberships of $\mu^{\left(2\right)}$, we have $\psi \left(\mu^{\left(1\right)},\mu^{\left(t\right)}\right)\geq \psi \left(\mu^{\left(2\right)},\mu^{\left(t\right)}\right)$ for any $\mu^{\left(t\right)}≽0$. According to this property, we can see that, if one fuzzy decision has more than one relative larger memberships, its corresponding consistency degree will be more probable larger than a fuzzy decision with only one relative larger membership. For example, given two fuzzy decisions $\mu^{\left(1\right)}={\left[0.1,\;0.7,\;0.3,\;0.2,0.2\right]}^{T}$ and $\mu^{\left(2\right)}={\left[0.1,\;0.7,\;0.8,\;0.2,0.2\right]}^{T}$, then for $\mu^{\left(t\right)}≽0$, we have $\psi \left(\mu^{\left(1\right)},\mu^{\left(t\right)}\right)\geq \psi \left(\mu^{\left(2\right)},\mu^{\left(t\right)}\right)$. This is because $\mu^{\left(2\right)}$ indicates that the classifier indicates that both class 2 and 3 are very probable to be the target class, and its consistency degree will be larger.*

With above consistency computation method, we can obtain a consistency matrix $A={\left[a_{t,k}\right]}_{T\times T}$ that contains pairwise consistency values of among the fuzzy decisions, in which $a_{t,k}=\psi \left(\mu^{\left(t\right)},\mu^{\left(k\right)}\right)$. It can be expected that, for a fuzzy decision $\mu^{\left(t\right)}$, if its consistency degrees $a_{t}=\left[a_{t,1},\ldots ,a_{t,T}\right]$ to other fuzzy decisions are relative lager values compared with other fuzzy decisions, then we can see it is more consistent to other fuzzy decisions, and its reliability degree should be higher. To achieve this goal, we use the eigenvalue decomposition method (EDM) [35] to compute the reliability weight of each fuzzy decision. In EDM, we want to compute the eigenvalues $\lambda_{1},\ldots ,\lambda_{T}$ and eigenvectors $w_{t},\ldots w_{T}$ of A, which satisfies the following condition:

(14) $$Aw_{t}=\lambda_{t}w_{t}$$

We can see that one eigenvalue corresponds to a unique eigenvector. The above EDM problem can be properly solved by using the well-known Singular Value Decomposition (SVD) method [35]. When all the eigenvalues and eigenvectors are obtained, we use the eigenvector $w^{s}$ that with the maximal eigenvalue $\lambda_{max}=\max\left\{\lambda_{1},\ldots ,\lambda_{T}\right\}$ as the decision weight vector. Note that the obtained eigenvector $w^{s}$ can not be directly used as reliability weight if it not normalized. Let $r={\left[r^{\left(t\right)}\right]}_{1\times T}$ be the normalized weight vector, and it is computed by

(15) $$r^{\left(t\right)}=a+b\frac{w_{t}^{s}-w_{min}^{s}}{w_{max}^{s}-w_{min}^{s}}$$

where a and b denote the lower bound and upper bound of the normalized weight respectively, $w_{max}^{s}$ and $w_{min}^{s}$ denote the maximal and minimal value of $w^{s}$, respectively. In this paper, we set a=0.6 and b=0.4; thus, we have $r^{\left(t\right)}\in \left[0.6,1\right].$

**Remark** **5.** *It has shown that eigenvector $w^{s}$ can be used as the representation of the importance of each vector in consistency matrix A [36,37]. More specifically, a relative larger value of $a_{t}$ will produce a relative larger eigenvalue of $w_{t}^{s}$. With this property, eigenvector $w^{s}$ can be used as the reliability degree of the fuzzy decisions. As mentioned above, a fuzzy decision with larger average consistency value corresponds to a relative larger eigenvalue, and it is more reliable compared with other fuzzy decisions.*

Next, we can combine all fuzzy decisions into a unified global one by using the obtained reliability weights, as given by

(16) $$\mu^{g}=\frac{1}{T}\overset{T}{\underset{t=1}{\sum }}r^{\left(t\right)}\mu^{\left(t\right)}$$

At last, the final decision is made by choosing the class with maximal global membership value, as given by

(17) $$c^{g}=\max\left\{\mu_{1}^{g},\ldots ,\mu_{N}^{g}\right\}$$

The above fusion process is suitable for classifying the feature data with different number of gait motion frames. It can be expected that, the fusion accuracy will be increased if the number of the fused decisions (or motion frame number *T*) is increased. In general, only several gait cycles (e.g., T≥3) will be sufficient to achieve robust fusion accuracy.

The detailed process of the proposed RWS rule is illustrated in the Algorithm 1. We first train the KELM classifier by using Equation (8). Note that, in the proposed method, only KELM is required to be trained, and RWS rule does not need to be trained and it can be directly used for combining the fuzzy decisions. Given a data with several new motion frames, we obtain the outputs of the KELM classifier, and transform the outputs into fuzzy decisions $\mu^{\left(1\right)},\ldots ,\mu^{\left(T\right)}$. Then, we compute the consistency matrix A by using the obtained fuzzy decisions. Subsequently, the eigenvalue decomposition process is conducted to matrix A, and the obtained eigenvector $w^{s}$ with the maximal eigenvalue is used for representing the reliability values of the fuzzy decisions. Subsequently, we normalize the eigenvector $w^{s}$ into a suitable interval and obtain the reliability vector $r={\left[r^{\left(t\right)}\right]}_{1\times T}$, and combine the fuzzy decisions by using a weighted sum combination operation. Finally, the classification result of all fuzzy decisions is chosen as the class with maximal global fuzzy membership.
**Algorithm 1** The proposed RWS decision fusion rule.**Input**: Motion frame data $\left(x^{\left(1\right)},y^{\left(1\right)},z^{\left(1\right)}\right),\ldots ;\left(x^{\left(T\right)},y^{\left(T\right)},z^{\left(T\right)}\right)$, RBF kernel parameter h, KELM regularization parameter λ;**Output**: Classification result $c^{g}$;1:**for**t=1,…,T2: Compute classification output $o^{\left(t\right)}$ by using Equation (10);3: Compute fuzzy decisions $\mu^{\left(t\right)}$ by using Equation (11);4:**end for**5:Compute fuzzy decision consistency matrix $A={\left[a_{t,k}\right]}_{T\times T}$ by using Equation (14);6:Compute eigenvalues $\lambda_{1},\ldots ,\lambda_{T}$ and eigenvectors $w_{t},\ldots w_{T}$ of consistency matrix $A={\left[a_{t,k}\right]}_{T\times T}$ by using eigenvalue decomposition;7:Find the eigenvector $w^{s}$ with the maximal eigenvalue $\lambda_{max}=\max\left\{\lambda_{1},\ldots ,\lambda_{T}\right\}$;8:Compute decision reliabilities $r={\left[r^{\left(t\right)}\right]}_{1\times T}$ by using Equation (16);9:Compute global fuzzy decision $\mu^{g}$ by using Equation (17);10:Obtain the final classification result $c^{g}$ by using Equation (18).

### 3.6. A Toy Example for Illustrating the Proposed RWS Rule

In this subsection we present a toy example to give a better understanding of the proposed RWS rule. Consider a gait motion recognition problem with 5 possible persons, and suppose that we have 10 consecutive motion frames, and the corresponding fuzzy decisions are shown in Table 1. In this example, the memberships of class 2 are randomly generated from interval (0.1, 0.8), and the memberships of other classes are randomly generated from interval (0.1, 0.4). We can see that, except $\mu^{\left(2\right)}$, the membership values of class 2 in other fuzzy decisions are actually not very large. In particular, we can see that in fuzzy decisions $\mu^{\left(1\right)},\;\mu^{\left(3\right)}$ and $\mu^{\left(6\right)}$, the classes with largest membership values are not class 2. It can be expected that their reliability degrees will be relative smaller than other fuzzy decisions.

Next, we compute the corresponding decision consistency matrix of the fuzzy decisions in Table 1, and the results are shown in Table 2, in which $A_{i}$ and $A^{\left(i\right)}$ denote the i-th column and i-th row, respectively. The corresponding fuzzy decisions are shown in Table 3. As expected, we can see the reliabilities of fuzzy decisions $\mu^{\left(1\right)},\;\mu^{\left(3\right)}$ and $\mu^{\left(6\right)}$ are the three smallest of the 10 fuzzy decisions, and reliability of $\mu^{\left(2\right)}$ is the largest. In particular, we can see that the reliabilities of $\;\mu^{\left(9\right)}$ and $\mu^{\left(10\right)}$, respectively. From this example, we can see that the obtained reliability weight of one fuzzy decision can reasonably reflect its overall consistency to other fuzzy decisions. Finally, with the obtained reliability weights, the global fuzzy decision can be obtained by Equation (17), which is $\mu^{g}=\left[0.11,\;0.27,\;0.16,\;0.14,\;0.14\right]$, which shows that the final decision is class 2.

## 4. Experimental Results

In this section, we test the performance of the proposed method by comparing it with some acknowledged baseline methods. The experiment mainly includes two parts: the first one is the classification performance comparison of the single motion frame, and the results of KELM, SVM, and random forest will be provided. The other one is the performance comparison results of the proposed RWS rule and other fusion rules, including sum rule, belief rule, weighted belief rule, product rule, and majority voting rule. As mentioned before, the system has 10 motion trackers, which are implemented on the lower body locations, and each body location has one or two trackers. For one body side, there are 5 trackers, and the trackers are implemented at the following three locations: thigh (one tracker), lower leg (one tracker at ankle, one tracker at shank), foot (one tracker at ankle, and one tracker at tiptoe). Since the system requires us to plant optical trackers on human bodies, it is better to reduce the number of trackers as much as possible. As such, we also want to clarify that whether it is possible for us to implement only one tracker on the lower leg and foot locations, thus we choose three trackers each body side, and six in total. With the above considerations, we will test the recognition performances of single motion frames and multiple motions with 10 and 6 motion trackers. All the experiments are conducted in a Windows 10 system with Intel i7 CPU, and 16 GB RAM, and the algorithm is developed in MATLAB 2020a platform.

### 4.1. Results of Single Motion Frame Classification

In this subsection, we test the classification performance of KELM on single motion frames. We first use 9595 motion frames as the training dataset, and the number of each of consecutive motion frames of each participant ranges from 82 to 179, and average number of each participant is about 122, and the corresponding number of gait cycles are about 3–5. We use another 4787 motion frames for the 76 participants, and the number of consecutive motion frames of each participant ranges from 45 to 89, or 1–3 gait cycles. We can see that test data size is about the 1/2 of the training data.

Except KELM, we also test the performance of support vector machines (SVM) and random forest, which are used in [26] and [27], respectively. The classification performance comparison results along with the parameter settings that can achieve the maximal classification accuracies are shown in Table 4 and Table 5. In Table 4, all the data collected from 10 motion trackers are used, while in Table 5, only 6 selected trackers are used, and they are located at front thigh, front knee, and ankle, both left foot and right foot. Since the collected data from 6 selected motion trackers are just a part of the data from all the 10 trackers, we can expect that its classification accuracy will be lower compared with the result of 10 motion trackers. For the random forest classifier, the number of template classification trees is set as 400, and the maximal tree split is set as 1500 or 2000. For the SVM classifier, the used kernel is RBF kernel, and the bandwidth parameter is $h=2^{-1}$ and $h=2^{-2}$ in Table 1 and Table 2, respectively. In KELM, the used kernel is also RBF kernel, the kernel bandwidth parameter and regularization parameter in Table 1 are set as $h=2^{3}$ and $\lambda =10^{4}$, respectively, and in Table 2 are $h=2^{1}$ and $\lambda =10^{4}$, respectively.

From Table 4 and Table 5, we can observe that, KELM achieves much better performances in both classification accuracy and training time compared with both SVM and random forest. More specifically, in Table 1, the classification of KELM achieves 27.38% and 33.11% higher accuracy compared with SVM and random forest, respectively. In Table 2, KELM achieves 24.37% and 31.75% classification accuracy improvement compared with SVM and random forest, respectively. In addition, SVM and random forest require more than 10 and 30 times of training time compared with KELM. The above results also indicate that, by adding more motion trackers, the classification accuracy of single motion frame will be increased. However, this practice will increase the deployment cost, and a relative lower classification accuracy for single motion frame will also have good fusion accuracy after combining multiple decisions of the frames.

### 4.2. Results of Multiple Decision Fusion

In this subsection, we demonstrate the classification performance of the proposed RWS rule by comparing it with several well-known decision fusion rules. The details of the compared rules as illustrated as follows:

(1) **Majority voting rule** [38]: Majority voting is a commonly used fusion rule for hard decisions, thus it is widely used in sensor fusion for its advantage in low data transmission amount. In this rule, we first obtain the hard decision of each motion frame, and then the final decision is made by choosing the class with maximal number of hard decisions. In this paper, if two class has the same voting number, the final decision is made by choosing the one with maximal average membership.

(2) **Sum rule** [38]: In this rule, the final fuzzy decision is simply computed by adding all the motion frame decisions, and the decision reliability is not considered. Given T motion frame fuzzy decisions $\mu^{\left(1\right)},\ldots ,\mu^{\left(T\right)}$, the global membership of class $c_{i}$ is computed as

(18) $$\mu_{i}=\frac{1}{T}\overset{T}{\underset{t=1}{\sum }}\mu_{i}^{\left(t\right)}$$

(3) **Product rule** [38]: similar to Naive Bayes fusion, in product rule the final fuzzy decision is obtained by the product of all the frame decisions. More specifically, the un-normalized global membership of class $c_{i}$ is computed as

(19) $$\mu_{i}=\overset{T}{\underset{t=1}{\prod }}(\mu_{i}^{\left(t\right)}+\delta )$$

where δ>0 is a very small constant to eliminate the influence of the memberships that are close to 0. In our experiment, we set $\delta =10^{-3}$.

(4) **Belief rule** [34]: Depmster-Shafer evidence theory is widely used in dealing with multiple decision fusion problems. The belief fusion rule is derived by combining multiple basic belief assignments (BBAs) by using the Dempster’s combinational rule, and it is also applicable in the multiple motion frame decision fusion problem. The un-normalized global BBA of class $c_{i}$ can be computed by

(20) $$\mu_{i}=\overset{T}{\underset{t=1}{\prod }}\frac{1}{\left(1-\mu_{i}^{\left(t\right)}+\delta \right)}$$

(5) **Reliability-weighted belief rule** [34]: The obtained decision reliability can also be used in computing the global BBA from the decisions of multiple motion frames. Similar to Equation (18), the un-normalized global BBA on class $c_{i}$ is given by

(21) $$\mu_{i}=\overset{T}{\underset{t=1}{\prod }}\frac{1}{\left(1-r^{\left(t\right)}\mu_{i}^{\left(t\right)}+\delta \right)}$$

**Figure 4:**
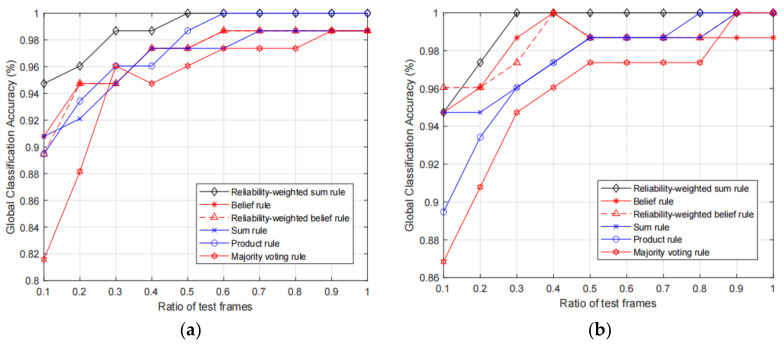
Comparison of fusion accuracies with 10 optical motion trackers, in which (**a**) and (**b**) show the results with original input features and calibrated input features, respectively.

**Figure 5:**
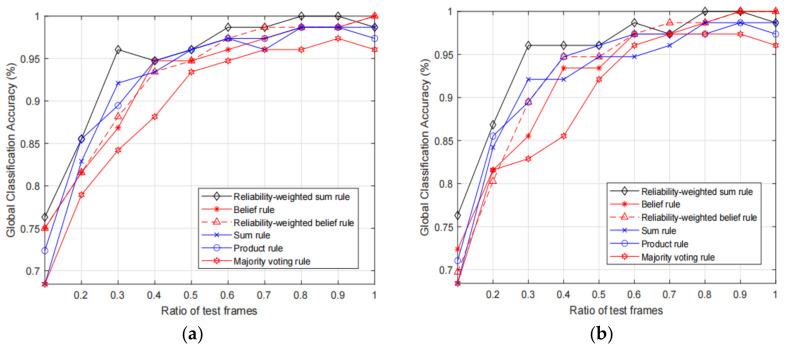
Comparison of fusion accuracies with 6 optical motion trackers, in which (**a**) and (**b**) show the results with original input features and calibrated input features, respectively.

Next, we test the classification accuracy of the fusion results of the proposed RWS rule and the baseline fusion rules with different KELM parameter settings. In addition, since the number of the test motion frames influences the classification, we also test the classification accuracies with increasing ratio of test motion frames, and ratio increases from 0.1 to 1. We test the fusion accuracies with the 10 trackers and 6 selected trackers, and the obtained results are shown in Figure 4 and Figure 5, respectively.

From the two figures, we can observe that the proposed RWS rule can achieve higher fusion accuracy compared with other rules. More specifically, we can see that, given the same ratio of the test frames, in most times the proposed RWS can achieve higher accuracies. However, it does not mean that the proposed rule will always be better than other rules. For example, in Figure 5b, we can see that belief rule and reliability-weighted belief rule achieve 100% accuracy, while the proposed rule only achieves 98.68%. As expected, the fusion accuracies of 10 trackers are much higher than the accuracies of 6 selected trackers. Therefore, if the deployment cost is acceptable, we can add more trackers to increase the classification accuracy. By comparing the results of Figure 4a,b we can observe that results with calibrated feature data are slightly better than the results with original non-calibrated data. This is because the classification performance of single motion frame is influenced by the unreliable features, and the performance will be increased if the unreliable features are calibrated.

## 5. Conclusions

In this paper, we have studied the gait recognition problem by using optical motion capture data, and we proposed a first-classification-then-fusion method, which includes the following four steps: feature extraction, unreliable feature detection, classification of single motion frame, and decision fusion of multiple frames. In particular, we proposed an RWS decision fusion rule to combine the fuzzy decision of the gait motions. The experimental results on 76 participants show that KELM achieves much higher classification accuracy and training efficiency compared with SVM and random forest in the single motion frame classification task, and they demonstrate that the proposed method RWS achieves better fusion accuracy compared with several existing fusion rules. Particularly, our results show that, with high-precision 3-D gait motion tracking data, the recognition method can achieve 100% accuracy when the full data of 10 optical trackers are used. 

It has to admit that, although the proposed method can achieve 100% recognition accuracy, the relative high implementation cost on both optical trackers and the capture cameras limits its application scenarios. On the other hand, our results indicate that the performance of other gait recognition systems, such as video surveillance, can be further improved if range sensor and depth sensor is integrated to enable measuring the distances of the captured persons. It has to be recognized that, though the proposed method can achieve very high recognition accuracy, it requires the implementation of optical motion trackers, which limits its practical application scenarios. 

Our future work is applying the proposed system in multiplayer motion tracking scenarios and test the performance with more complex trajectories. In this way, the system can support several players in the tracking field at the same time, which will greatly enhance its capability and efficiency.

## Figures and Tables

**Table 1:** The fuzzy decisions.

	Class 1	Class 2	Class 3	Class 4	Class 5
$\mu^{\left(1\right)}$	0.23	0.24	0.34	0.13	0.40
$\mu^{\left(2\right)}$	0.32	0.71	0.39	0.23	0.32
$\mu^{\left(3\right)}$	0.10	0.29	0.19	0.39	0.18
$\mu^{\left(4\right)}$	0.19	0.52	0.31	0.26	0.34
$\mu^{\left(5\right)}$	0.14	0.40	0.36	0.31	0.13
$\mu^{\left(6\right)}$	0.13	0.33	0.37	0.19	0.23
$\mu^{\left(7\right)}$	0.16	0.57	0.13	0.31	0.37
$\mu^{\left(8\right)}$	0.20	0.36	0.11	0.35	0.19
$\mu^{\left(9\right)}$	0.22	0.50	0.15	0.11	0.19
$\mu^{\left(10\right)}$	0.26	0.58	0.36	0.33	0.14

**Table 2:** The consistency matrix of the fuzzy decisions.

	$A_{1}$	$A_{2}$	$A_{3}$	$A_{4}$	$A_{5}$	$A_{6}$	$A_{7}$	$A_{8}$	$A_{9}$	$A_{10}$
$A^{\left(1\right)}$	0.40	0.53	0.28	0.44	0.34	0.35	0.40	0.29	0.31	0.42
$A^{\left(2\right)}$	0.53	0.92	0.46	0.72	0.59	0.54	0.69	0.50	0.57	0.76
$A^{\left(3\right)}$	0.28	0.46	0.31	0.39	0.34	0.30	0.39	0.31	0.27	0.42
$A^{\left(4\right)}$	0.44	0.72	0.39	0.59	0.47	0.44	0.57	0.41	0.44	0.60
$A^{\left(5\right)}$	0.34	0.59	0.34	0.47	0.43	0.38	0.44	0.35	0.34	0.52
$A^{\left(6\right)}$	0.35	0.54	0.30	0.44	0.38	0.36	0.40	0.30	0.31	0.46
$A^{\left(7\right)}$	0.40	0.69	0.39	0.57	0.44	0.40	0.60	0.43	0.44	0.57
$A^{\left(8\right)}$	0.29	0.50	0.31	0.41	0.35	0.30	0.43	0.34	0.31	0.44
$A^{\left(9\right)}$	0.31	0.57	0.27	0.44	0.34	0.31	0.44	0.31	0.36	0.46
$A^{\left(10\right)}$	0.42	0.76	0.42	0.60	0.52	0.46	0.57	0.44	0.46	0.66

**Table 3:** The reliabilities of the fuzzy decisions.

	$\mu^{\left(1\right)}$	$\mu^{\left(2\right)}$	$\mu^{\left(3\right)}$	$\mu^{\left(4\right)}$	$\mu^{\left(5\right)}$	$\mu^{\left(6\right)}$	$\mu^{\left(7\right)}$	$\mu^{\left(8\right)}$	$\mu^{\left(9\right)}$	$\mu^{\left(10\right)}$
**Reliability** (Unnormalized)	0.26	0.44	0.24	0.36	0.29	0.27	0.35	0.26	0.27	0.37
**Reliability**	0.37	1.00	0.30	0.70	0.48	0.39	0.66	0.35	0.39	0.75

**Table 4:** Classification accuracy with all 10 motion trackers.

Classifier	Accuracy (%)	Train Time(s)	Parameter Settings
Random Forest [27]	50.44	74.62	Number of Trees = 400, Maxsplit = 2000
SVM [26]	56.17	27.88	RBF Kernel, $h=2^{-3}$
KELM	83.55	2.46	RBF Kernel, $h=2^{4}$, $\lambda =10^{5}$

**Table 5:** Classification accuracy with 6 selected motion trackers.

Classifier	Accuracy (%)	Train Time(s)	Parameter Settings
Random Forest [27]	36.42	45.77	Number of Trees = 400, Maxsplit = 1500
SVM [26]	43.80	18.19	RBF Kernel, $h=2^{-2}$
KELM	68.17	2.61	RBF Kernel, $h=2^{1}$, $\lambda =10^{7}$

## Data Availability

Not applicable.
